# Supplementary material for: The Draft Genome of Kochia scoparia and the Mechanism of Glyphosate Resistance via Transposon-Mediated EPSPS Tandem Gene Duplication
Source: Genome Biol Evol. 2019 Sep 13;11(10):2927–40. doi: 10.1093/gbe/evz198 (PMC6808082; doi:10.1093/gbe/evz198)
Supplement: evz198_Supplementary_Data [file evz198_supplementary_data.zip › Supporting Information ksEPSPS CNV_R1.pdf]

**Supporting Information**

**Methods**

DNA Extraction

For shotgun genome Illumina sequencing of the two lines, DNA was extracted from samples using a modified CTAB extraction protocol described in Doyle (1991). First, 500 µl of extraction buffer (100 mM tris, 1.4 M NaCl, 20 mM EDTA, pH 8.0, 2% CTAB, 0.3% mercaptoethanol) with 5 mg polyvinylpyrrolidone (PVP) was mixed with the tissue aliquots. The suspension was homogenized and incubated at 60 °C for 15 min. Next, 500 µl of chloroform:isoamyl alcohol (24:1) was added and the tubes were gently agitated on an orbital mixer for 15 min. The tubes were then centrifuged at 8000 rcf for 15 min and the top, aqueous phase was moved to a new tube. One µl of RNase A was added and incubated at 37 °C for 1 hour. The chloroform:isoamyl alcohol separation was performed again and the aqueous phase retained again. DNA was then precipitated by adding 1/10 volume 5M sodium acetate, pH 8 and three volumes of 100% ethanol. The samples were then centrifuged at 10,000 rcf for 10 min. The supernatant was poured off and the resulting pellet was rinsed with 70% ethanol and then allowed to dry. The pellet was re-suspended in 100 µl of water, checked for concentration and purity on a Nanodrop T1000.

For large-fragment, genomic PacBio sequencing of the glyphosate-susceptible line, the CTAB protocol was further modified to obtain more DNA of sufficiently large size (>10kb). Approximately 1 g of finely chopped kochia young leaf tissue was added to 50 ml conical tubes. To this tissue 15 ml of CTAB extraction buffer and 60 µg of PVP were added, mixed, and allowed to incubate for 30 min at 50 °C. The tubes were then centrifuged at 3600 rcf for 10 min. The liquid phase was separated into a new tube and 15 mL of chloroform:isoamyl alcohol (24:1)

was added and mixed by inversion. They were then centrifuged at 3600 rcf for 10 min more and the upper phase transferred to a new tube. To this 4 µl of RNase A was added and incubated for 30 min at 37 °C. The chloroform:isoamyl alcohol separation was repeated and the final aqueous phase collected. The DNA was precipitated by adding 3 volumes of EtOH and 0.5 volume of NaCl 5M. The tubes were then incubated at -20 °C for 30 min, centrifuged at 3600 rcf for 10 min, and the pellet washed with 70% ethanol. The final pellets were dried and re-suspended in 2 ml of Tris-EDTA buffer. The DNA was further purified using the Genomic DNA Clean & Concentrator™-10 kit by Zymo, following the recommended protocol. The DNA was checked for quality using a NanoDrop 2000c and quantified using Qubit.

**References**

Doyle J (1991) DNA protocols for plants–CTAB total DNA isolation. In ‘Molecular techniques in taxonomy’. (Eds GM Hewitt, A Johnston) pp. 283–293 Springer: Berlin.

## SUPPORTING INFORMATION FIGURES

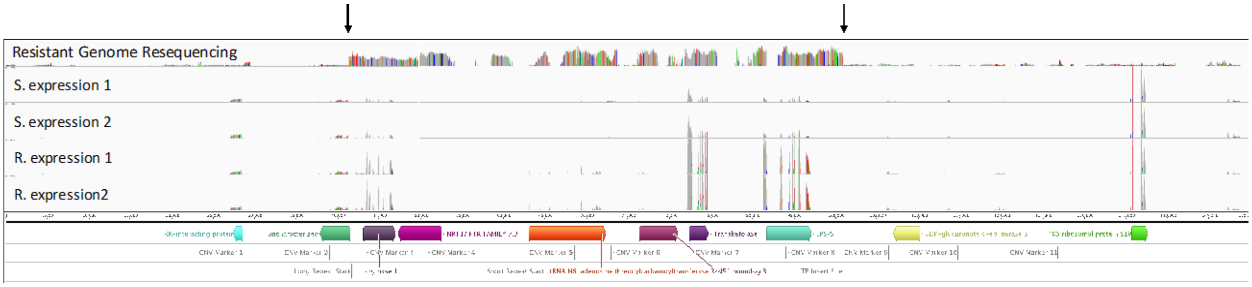


Sup. Figure 1. The first 150,000 bp from the *EPSPS* contig from the kochia genome assembly. Predicted genes are represented by the multicolored blocks and labeled with text of the corresponding color. The locations of copy number qPCR markers are indicated, as well as the beginning of the 56.1 kb repeat, 32.7 kb repeat, and MGE insert site. The beginning and end of the duplication are indicated with black arrows. Alignments of RNA-Seq Illumina data from two resistant and two susceptible individuals are indicated as well as whole genome resequencing data from the resistant line.

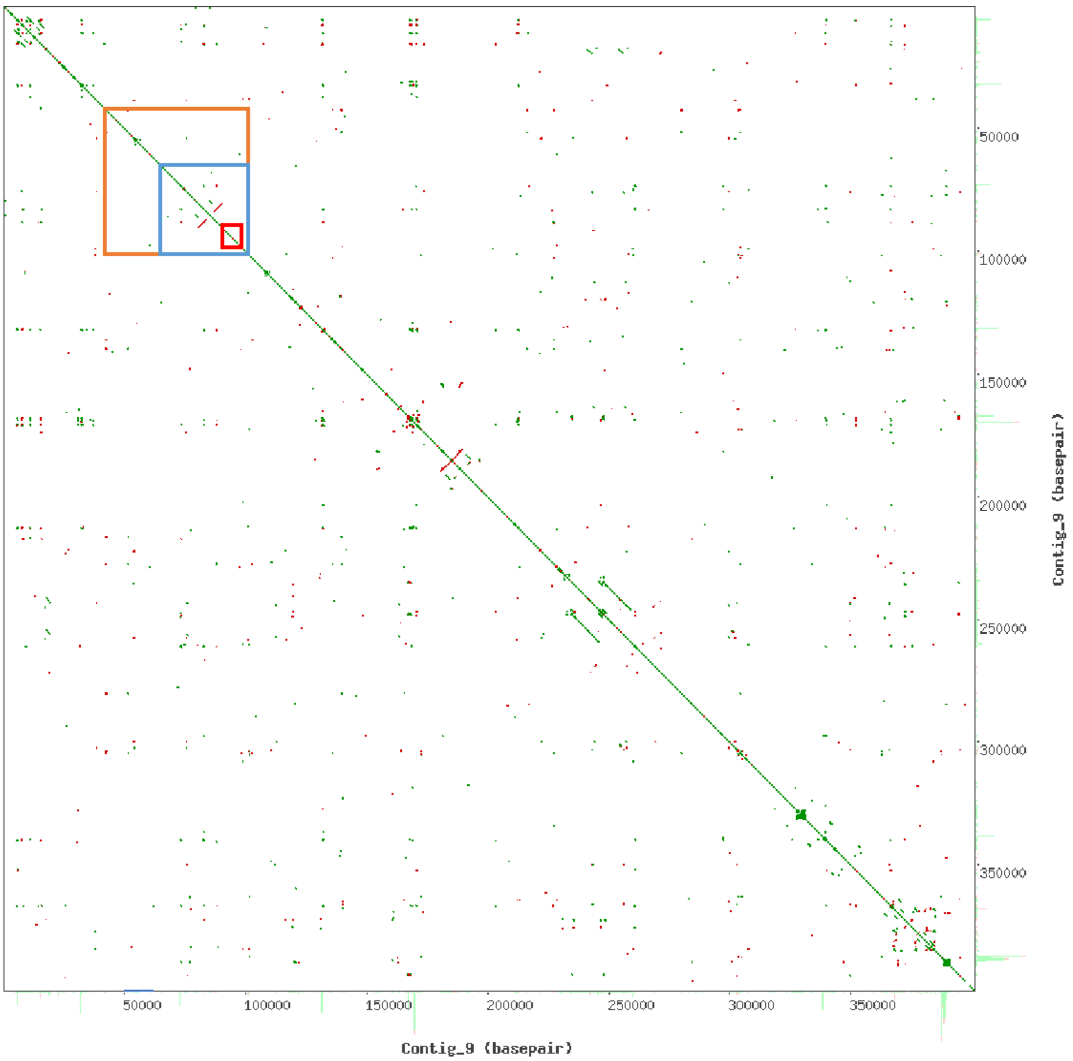


Sup. Figure 2. A self-alignment of the *EPSPS* contig from the kochia genome assembly. The location of the *EPSPS* gene is indicated with a red box. The 56.1 kb repeat is indicated with an orange box, the 32.7 kb repeat is indicated using a blue box.

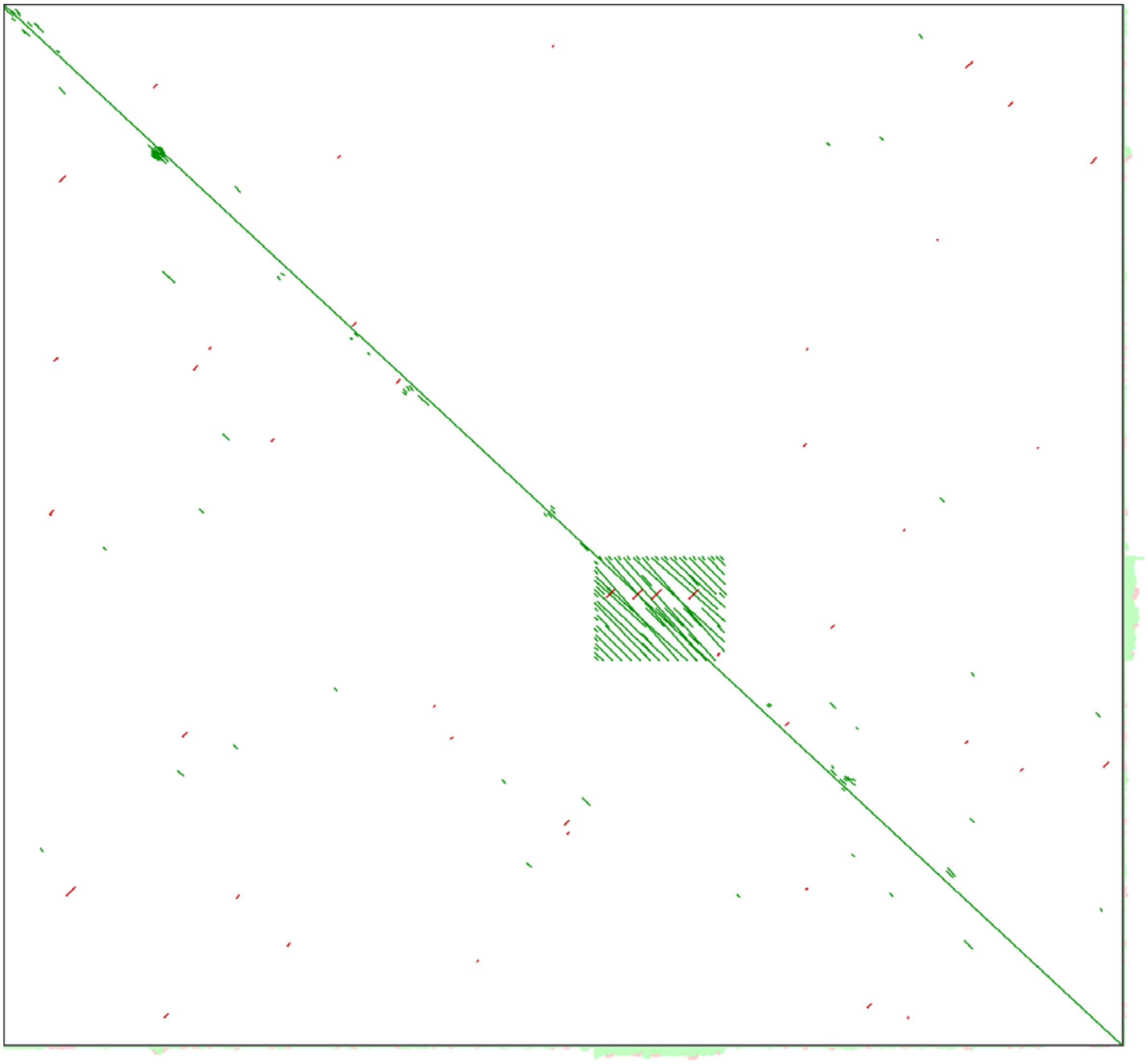

Sup. Figure 3. An alignment of the MGE from the BACs of the resistant plant (sequence >Resistant_MGE in the file all_fasta.txt) and the MGE assembled from the PacBio data from the susceptible genome assembly (sequence >Susceptible_MGE in the file all_fasta.txt).

## SUPPORTING INFORMATION TABLES

Sup. Table 1. Primers for qPCR markers for determining copy number at multiple locations near the *EPSPS* gene and qPCR markers for determining copy number of 56.1 kb repeats, 32.7 kb repeats, and the MGE. Base-pair coordinates of PCR amplicons are given relative to their position in the contig from the susceptible genome assembly.

| Primer name | Primer sequence | Melting Temp (°C) | GC Content (%) | Base-Pair Start/Stop |
|---|---|---|---|---|
| 1 | 5’-CATAGGTTGAGGGTGGACTTTC-3’ | 55.2 | 50 | 28,602 |
| 1 | 5’-GGTGTTTGTTTGACCACCTTTC-3’ | 54.8 | 45.5 | 28,712 |
| 2 | 5’-TTCTGCCTCAGCAAACATACT-3’ | 54.3 | 42.9 | 39,028 |
| 2 | 5’-CATGGTCACTTTGTGTGTCATTAG-3’ | 54.2 | 41.7 | 39,127 |
| 3 | 5’-CTCGGAAAGGATGGAAGAATG-3’ | 53.2 | 47.6 | 43,248 |
| 3 | 5’-GTTATGTCCTGTCTTCTGTGTG-3’ | 53.2 | 45.5 | 43,408 |
| 4 | 5’-TTTCGCTTTCCGAGGTAATAG-3’ | 52.4 | 42.9 | 50,680 |
| 4 | 5’-CAACTAACACGAACATTGTGTC-3’ | 52.2 | 40.9 | 50,833 |
| 5 | 5’-TCGAAGCCTGACATTAGATTAG-3’ | 51.9 | 40.9 | 68,546 |
| 5 | 5’-CTCTTTGTACCTGATCCCATC-3’ | 52.5 | 47.6 | 68,700 |
| 6 | 5’-CTCCTCCTCCCTCCTAATATC-3’ | 53 | 52.4 | 73,024 |
| 6 | 5’-CTTGTTTCCTCCTCTCGTTC-3’ | 52.9 | 50 | 73,154 |
| 7 | 5’-TCATCCCTTTCTCTCTCCTC-3’ | 52.9 | 50 | 82,513 |
| 7 | 5’-GATAAGTCCGTCAACACGATC-3’ | 53.1 | 47.6 | 82,687 |
| 8 | 5’-GACATCCTGTCATGGAGTAAG-3’ | 52.4 | 47.6 | 94,023 |
| 8 | 5’-CCTAAATAAACCGGAAGCAATC-3’ | 51.8 | 40.9 | 94,172 |
| 9 | 5’-TCAACACCCAACTCACATCTC-3’ | 54.7 | 47.6 | 106,488 |
| 9 | 5’-TAGAAGCACAGGAGAGAGAGAA-3’ | 54.5 | 45.5 | 106,610 |
| 10 | 5’-GGCATGTGGAGAAGATGTATAG-3’ | 52.7 | 45.5 | 114,766 |
| 10 | 5’-CTTTGTTGGTTCAATTGGAGG-3’ | 52.2 | 42.9 | 114,942 |
| 11 | 5’-TCGGATCCCTTAGATACACTAC-3’ | 52.8 | 45.5 | 126,791 |
| 11 | 5’-GTTACCTGTCTTGAGCAGTG-3’ | 53.1 | 50 | 126,950 |
| Repeat Type-FP | 5’-GACGGAAATACCCTCAATATAGACA-3’ | 54.0 | 40.0 | N/A |
| 56.1kb RP | 5’-ACGCCCAAGATGTACATTGATA-3’ | 54.0 | 40.9 | N/A |
| 32.7kb RP | 5’-CATGCCTTTGATGTCCAAGTTT-3’ | 54.1 | 40.9 | N/A |
| Fhy3/FAR1 FP | 5’-GAAGATAGCGAGACGTTTGAG-3’ | 53.0 | 47.6 | N/A |
| Fhy3/FAR1 RP | 5’-CGGCTTGATCGGTTAAGATAC-3’ | 53.2 | 47.6 | N/A |

Sup. Table 2. A statistical summary of the kochia genome assembly.

| Metric | Count | Percentage |
|---|---|---|
| Number of scaffolds | 19,671 | |
| Total size of scaffolds (bp) | 711,356,803 | |
| Longest scaffold (bp) | 770,912 | |
| Shortest scaffold (bp) | 897 | |
| Scaffold length/genome size | | 83.70% |
| | | |
| Number of scaffolds > 1K nt | 19,594 | 99.6% |
| Number of scaffolds > 10K nt | 14,701 | 74.7% |
| Number of scaffolds > 100K nt | 1,286 | 6.5% |
| Mean scaffold size (bp) | 36,163 | |
| N50 scaffold length (bp) | 61,675 | |
| | | |
| %A | | 28.8% |
| %C | | 16.4% |
| %G | | 16.4% |
| %T | | 28.5% |
| %N | | 9.5% |
| | | |
| Num. of contigs | 61,353 | |
| Num. of contigs in scaffolds | 54,776 | |
| Total size of contigs | 643,547,114 | |

Sup. Table 3. A statistical summary of predicted genes in the kochia genome.

| Metric | Count | Percentage |
|---|---|---|
| Proteome | | |
| Total Length of Proteome aa | 14,859,659 | |
| Longest Protein | 5,817 | |
| Number of Transcripts > 500 aa | 8,158 | |
| Number of Transcripts > 1,000 aa | 1204 | |
| Mean Protein Size | 313 | |
| Median Protein Size | 234 | |
| | | |
| Transcriptome | | |
| Number of Coding Gene Models (Maker) | 47,414 | |
| Total Length of Transcripts | 44,695,962 | |
| Longest Transcript | 17,454 | |
| Number of Transcripts > 500 nt | 30,953 | 65.3% |
| Number of Transcripts > 1K nt | 16,209 | 34.2% |
| Number of Transcripts > 10K nt | 12 | 0.0% |
| Mean Transcript size | 943 | |
| Median Transcript size | 702 | |
| N50 transcript length | 1,311 | |
| L50 transcript count | 10,590 | |
| scaffold %A | | 27.9% |
| scaffold %C | | 22.1% |
| scaffold %G | | 22.1% |
| scaffold %T | | 27.8% |
| scaffold %N | | 0.1% |
| | | |
| Annotation | | |
| Number of Proteins with Blast Hit (DataBase) | 29,730 | 62.70% |
| Number of Proteins with InterPro Domain | 38,779 | 81.79% |

Sup. Table 4. Assessing the kochia genome assembly and annotation completeness with BUSCO.

| Metric | Count | Percentage |
|---|---|---|
| # of Ultra-conserved Genes Searched For | 2121 | |
| # Ultra-conserved Single Genes Found | 1437 | 67.8% |
| # Ultra-conserved Duplicated Genes Found | 53 | 2.5% |
| # Ultra-conserved Partial Genes Found | 172 | 8.1% |
| Total Ultra-conserved Genes Found | 1662 | 78.4% |
| # Ultra-conserved Genes Missing | 459 | 21.6% |

Sup. Table 5. Analysis of repetitive content in the kochia genome using RepeatMasker.

| Interspersed repeat elements | Number | Length (BP) | % of Assembly |
|---|---|---|---|
| Retroelements | 66,766 | 38,463,923 | 5.41% |
| SINEs: | 178 | 26,154 | 0% |
| Penelope | 8 | 787 | 0% |
| LINEs: | 12,579 | 4,566,194 | 0.64% |
| CRE/SLACS | 199 | 137,148 | 0.02% |
| L2/CR1/Rex | 0 | 0 | 0% |
| R1/LOA/Jockey | 0 | 0 | 0% |
| R2/R4/NeSL | 0 | 0 | 0% |
| RTE/Bov-B | 3,377 | 1,311,037 | 0.18% |
| L1/CIN4 | 9,011 | 3,123,321 | 0.44% |
| LTR elements: | 54,009 | 33,871,575 | 4.76% |
| BEL/Pao | 0 | 0 | 0% |
| Ty1/Copia | 22,611 | 15,381,646 | 2.16% |
| Gypsy/DIRS1 | 30,306 | 18,264,655 | 2.57% |
| Retroviral | 0 | 0 | 0% |
| DNA transposons | 27,584 | 5,607,206 | 0.79% |
| hobo-Activator | 10,360 | 1,763,567 | 0.25% |
| Tc1-IS630-Pogo | 3,368 | 819,160 | 0.12% |
| En-Spm | 0 | 0 | 0% |
| MuDR-IS905 | 0 | 0 | 0% |
| PiggyBac | 0 | 0 | 0% |
| Tourist/Harbinger | 1,508 | 538,707 | 0.08% |
| Other (Mirage, P-element, Transib) | 2 | 74 | 0% |
| Rolling-circles | 0 | 0 | 0% |
| Unclassified: | 1,535 | 392,232 | 0.06% |
| Total interspersed repeats: | | 44,463,361 | 6.25% |

| Other Repeats | Number | Length (BP) | % of Assembly |
|---|---|---|---|
| Small RNA: | 948 | 223,307 | 0.03% |
| Satellites: | 256 | 22,750 | 0% |
| Simple repeats: | 261,069 | 14,664,544 | 2.06% |
| Low complexity: | 58,540 | 3,248,675 | 0.46% |
| Total Other repeats: | | 18,159,276 | 2.55% |

Sup. Table 6. Annotation (.bed) file for >BAC_Assembly_of_EPSPS_CNV sequence in all_fasta.txt; indicates locations of repeat types assembled from kochia BAC sequences.

| | | | |
|---|---|---|---|
| Resistant_CNV | 1 | 49403 | Upstream_region |
| Resistant_CNV | 49804 | 65964 | MGE_For |
| Resistant_CNV | 66364 | 122102 | TYPE1 |
| Resistant_CNV | 122503 | 138127 | MGE_For |
| Resistant_CNV | 138528 | 170805 | TYPE2 |
| Resistant_CNV | 171206 | 186830 | MGE_For |
| Resistant_CNV | 187230 | 242968 | TYPE1 |
| Resistant_CNV | 243368 | 258993 | MGE_For |
| Resistant_CNV | 259393 | 275018 | MGE_Rev |
| Resistant_CNV | 275418 | 331156 | TYPE_3(Type1_Rev) |
| Resistant_CNV | 331556 | 346817 | MGE_Rev |
| Resistant_CNV | 347589 | 365421 | TYPE_4 |
| Resistant_CNV | 365421 | 398519 | TYPE_5 |
| Resistant_CNV | 398919 | 414544 | MGE_For |
| Resistant_CNV | 414944 | 429317 | Downstream_Region |
